# Whole-genome sequence analysis reveals the circulation of multiple SARS-CoV-2 variants of concern in Nairobi and neighboring counties, Kenya between March and July 2021

**DOI:** 10.1186/s12985-022-01895-y

**Published:** 2022-11-08

**Authors:** Samoel Ashimosi Khamadi, Silvanos Opanda, Samwel Lifumo Symekher, Samson Konongoi Limbaso, Solomon Langat, Josyline Kaburi Cirindi, Milkah Mwangi, Nicholas Mwikwabe, Seth Okeyo, Edith Koskei, James Mutisya, Samwel Owaka, Albert Nyunja, Hellen Koka, Meshack Wadegu, Esther Chitechi, Rachel Achilla, Janet Masitsa Majanja, Lucy Kanyara, Evans Amukoye, Wallace Bulimo

**Affiliations:** 1 Centre for Virus Research, Kenya Medical Research Institute (KEMRI), P.O. Box 54628-00200, Nairobi, Kenya; 2 Centre for Traditional Medicine and Drug Research, Kenya Medical Research Institute (KEMRI), Nairobi, Kenya; 3 Centre for Biotechnology Research and Development, Kenya Medical Research Institute (KEMRI), Nairobi, Kenya; 4 Centre for Respiratory Diseases Research, Kenya Medical Research Institute (KEMRI), Nairobi, Kenya

**Keywords:** SARS-CoV-2, Coronaviruses, Lineage, Kenya

## Abstract

The emergence and rapid spread of SARS-CoV-2 variants of concern (VOC) have been linked to new waves of COVID-19 epidemics occurring in different regions of the world. The VOC have acquired adaptive mutations that have enhanced virus transmissibility, increased virulence, and reduced response to neutralizing antibodies. Kenya has experienced six waves of COVID-19 epidemics. In this study, we analyzed 64 genome sequences of SARS-CoV-2 strains that circulated in Nairobi and neighboring counties, Kenya between March 2021 and July 2021. Viral RNA was extracted from RT-PCR confirmed COVID-19 cases, followed by sequencing using the ARTIC network protocol and Oxford Nanopore Technologies. Analysis of the sequence data was performed using different bioinformatics methods. Our analyses revealed that during the study period, three SARS-CoV-2 variants of concern (VOC) circulated in Nairobi and nearby counties in Kenya. The Alpha (B.1.1.7) lineage predominated (62.7%), followed by Delta (B.1.617.2, 35.8%) and Beta (B.1.351, 1.5%). Notably, the Alpha (B.1.1.7) VOC were most frequent from March 2021 to May 2021, while the Delta (B.1.617.2) dominated beginning June 2021 through July 2021. Sequence comparisons revealed that all the Kenyan viruses were genetically similar to those that circulated in other regions. Although the majority of Kenyan viruses clustered together in their respective phylogenetic lineages/clades, a significant number were interspersed among foreign strains. Between March and July 2021, our study's findings indicate the prevalence of multiple lineages of SAR-CoV-2 VOC in Nairobi and nearby counties in Kenya. The data suggest that the recent increase in SARS-CoV-2 infection, particularly in Nairobi and Kenya as a whole, is attributable to the introduction and community transmission of SARS-CoV-2 VOC among the populace. In conclusion, the findings provide a snapshot of the SARS-CoV-2 variants that circulated in Kenya during the study period.

## Introduction

Severe acute respiratory syndrome coronavirus 2 (SARS-CoV-2) first emerged in Wuhan, China on 8th December 2019 [1] and rapidly spread across the world, causing coronavirus disease of 2019 (COVID-19) pandemic [1–3]. Until now, the SARS-CoV-2 continues to circulate in many countries, causing multiple waves of COVID-19 epidemics [4]. Indeed, as of 21st July 2022, > 564 million people have been infected worldwide, with > 6 million deaths reported, with Kenya registering 336,904 cases and 5668 deaths [5]. Since its first appearance, the SARS-CoV-2 has undergone significant numbers of mutations over time, giving rise to multiple genetic variants, some of which have beneficial host adaptive fitness [4, 6–11]. The emerging SARS-CoV-2 variants have been described as variants of interest (VOI) and variants of concern (VOC) [10, 12]. Certainly, considerable attention has been focused on the latter as they harbor genetic attributes associated with increased transmissibility, increased virulence, and reduced effectiveness of public health countermeasures [4, 8–10].

Five major SARS-CoV-2 lineages, Alpha (B.1.1.7), first reported in the United Kingdom; Beta (B.1.351), first reported in South Africa; Delta (B.1.617.2) first seen in India; Gamma (P.1) first seen in Japan/Brazil; and Omicron (B.1.1.529) first reported in South Africa have been identified as variants of concern [4, 8–10, 13–16]. They possess point mutations in the receptor-binding domain (RBD) of the spike protein, which enhances binding affinity to the human angiotensin-converting enzyme 2 (ACE2) receptor. The Alpha variants contain asparagine to tyrosine mutation (N501Y) in the spike protein RBD; Beta (N501Y, K417N,& E484K); Delta (L452R) while the Gamma P1variant has acquired 17 mutations of which K417N, E484K, and N501Y are contained in the spike protein [9, 10, 17, 18]. The Omicron variant is heavily mutated, with thirty amino acid variations within the surface spike protein, mostly affecting the receptor-binding domain (RBD) [19]. However, some of the concerning mutations including the N501Y, D614G, K417N, and T478K are present among the previous variants of concern [14].

After the emergence and rapid transmission, accompanied by devastating disease outcomes in the source countries, the SARS-CoV-2 VOC have spread to other regions, causing multiple waves of COVID-19 outbreaks [16, 18, 20]. Kenya in particular has experienced multiple waves of SARS-CoV-2 infection since the detection the first case in the country [21]. The first peak in SARS-CoV-2 infection occurred in August 2020 and was attributed to the relaxation of public health restriction measures while the second and third waves occurred in November 2020 and March 2021 respectively, seemingly due to the emergence of genetic variants of concern [21]. However, after a short lull and beginning in June 2021, a surge in SARS-CoV-2 infection was observed in Kenya [21], especially in the western city of Kisumu and the capital city of Nairobi, yet genomic characteristics of these devastating variants have not been determined.

Whole-genome sequence analysis is recognized for its importance in providing insights into virus transmission patterns and evolutionary dynamics [22]. However, aside from the study by Githinji et al. [23] describing the importation and local transmissions of SARS-CoV-2 strains, full genome studies involving SARS-CoV-2 variants circulating in Kenya remain scanty. The current study sought to characterize the full genome sequences of SARS-CoV-2 that circulated in Nairobi and neighboring counties in Kenya between March and July 2021, during the third COVID-19 wave in the country, to describe the variants and classify them into VOC lineages. Because Nairobi is Kenya's travel hub, the data provided here provide a glimpse of the introductions and temporal dynamics of SARS-CoV-2 VOC circulation in Kenya.

## Methods

### Sampling

Respiratory samples were collected from persons who presented for voluntary COVID-19 testing at the Kenya Medical Research Institute (KEMRI). The testing was carried out as part of the Kenya Ministry of Health’s (MoH) national response to control COVID-19. Persons presenting for a COVID-19 test filled out a questionnaire capturing their demographic and residence information.

### RNA extraction, genome amplification, and sequencing

Genomic surveillance for the SARS-CoV-2 was conducted between March 2021 and July 2021 to investigate the presence and spread of the SARS-CoV-2 variants of concern (VOC) in Nairobi and neighboring counties, Kenya. Samples received at KEMRI’s Sample Management and Repository Facility (SMRF) were tested for COVID-19 using the real-time RT-PCR assay. Viral RNA was extracted from nasopharyngeal/oropharyngeal swab specimens using the QIAamp Viral RNA Mini Kit (QIAGEN, Germany) following the manufacturer's protocol. SARS-CoV-2 real-time RT-PCR assay was performed using different commercial kits viz. DaAn Gene Detection kit for 2019-nCoV (DaAnGene, China), RADI COVID-19 Detection kit (KHMedical, South Korea) and SD Biosensor Covid 19 RT PCR Test Kit (SD BIOSENSOR Republic of Korea), according to the World Health Organization’s (WHO) guidelines [24]. Selected samples that tested positive with threshold (Ct) values < 30, for the E & RdRp or S & RdRp or ORF1ab & N target genes were selected and RT-PCR and SARS-CoV-2 genome sequencing performed following ARTIC’s network protocol and Oxford Nanopore Technologies, as previously described [25, 26]. Viral genome assembly and variant assignment were carried out based on ARTIC Network bioinformatics protocol [27, 28]. Mutational analysis was performed using various web tools including CoVsurver [29], CoV-GLUE [30], and Nexclade [31].

### Phylogenetic analysis

The SARS-CoV-2 genome sequences were grouped into genetic lineages based on the Phylogenetic Assignment of Named Global Outbreak LiNeages (PANGOLIN) [32] and NextStrain [33] classification methods. The genome sequences were analyzed relative to NC_045512.2-Wuhan-Hu-1 and those obtained from other regions between March 2021 and July 2021. The latter were sampled from the coronavirus data collection of the Global initiative on sharing all influenza data (GISAID) [34] based on phylogenetic placement and GISAID’s BLAST tool within the EpiCoV™ browser [35]. Sequences with a match quality of ≥ 0.990 were selected. Low-quality, divergent, and incomplete genomes (< 29,000 bp) were filtered out, leaving 135 reference genomes. Multiple sequence alignment of the local SARS-CoV-2 genomes alongside those of foreign strains sampled from the GISAID [34], resulting in a final dataset of 199 sequences was performed using MAFFT software v7 [36]. The 3’ and 5’ untranslated regions were trimmed using Unipro UGENE v39.0 software [37], followed by masking of the three known sequencing error-prone regions (13,402, 24,389, and 24,390). The best-fit substitution model for the dataset was determined using ModelFinder [38]. A maximum-likelihood tree was re-constructed with IQ-TREE v1.6.12 [39] utilizing the GTR + F + R2 model. Branch support was established by ultrafast bootstrap and SH-like approximate likelihood ratio test (SH-aLRT) with 1000 replicates. The consensus tree was annotated and displayed with the interactive tree of life v4 (iTOL) [40].

## Results

**Fig. 1:**
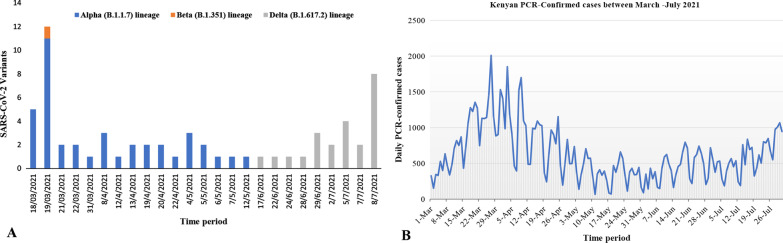
Distribution of SARS-CoV-2 variants and cases detected in Nairobi and Kenya respectively, between March–July 2021. **A** The bar graph showing frequency of SARS-CoV-2 lineages detected among PCR-confirmed samples sampled in Nairobi County during the study period. The Alpha, Beta, and Delta lineages are shown in blue, orange and grey, respectively. **B** Graph showing the daily distribution of PCR-confirmed SARS-CoV-2 cases in Kenya between March–July 2021. The *y*-axis indicates the frequency of detections, while the *x*-axis represents the time-period of each day of the sampling period

Sixty-four SARS-CoV-2 genome sequences were obtained over the study period. Of these, forty-one [41] were found to belong to the Alpha (B.1.1.7) lineage, twenty-two [22] to Delta (B.1.617.2), and one (1) to Beta (B.1.351). The majority of the individuals from which viral sequences were obtained were male (n = 43; 68.3%). The patients’ ages ranged from 16 to 69 years. Markedly, the Alpha and Beta SARS-CoV-2 genomes were recovered from the samples collected between March and May 2021, while viruses of the Delta lineages originated from samples collected during June and July 2021. From these data sets it can be concluded that the sequenced samples from Kenya during this period represent only 0.1% or less of the co-circulating SARS-CoV-2 causing confirmed infections. Figure 1 summarizes the distribution of SARS-CoV-2 variants detected in the study, as well as the PCR-confirmed cases in the country from March – July 2021.

**Fig. 2:**
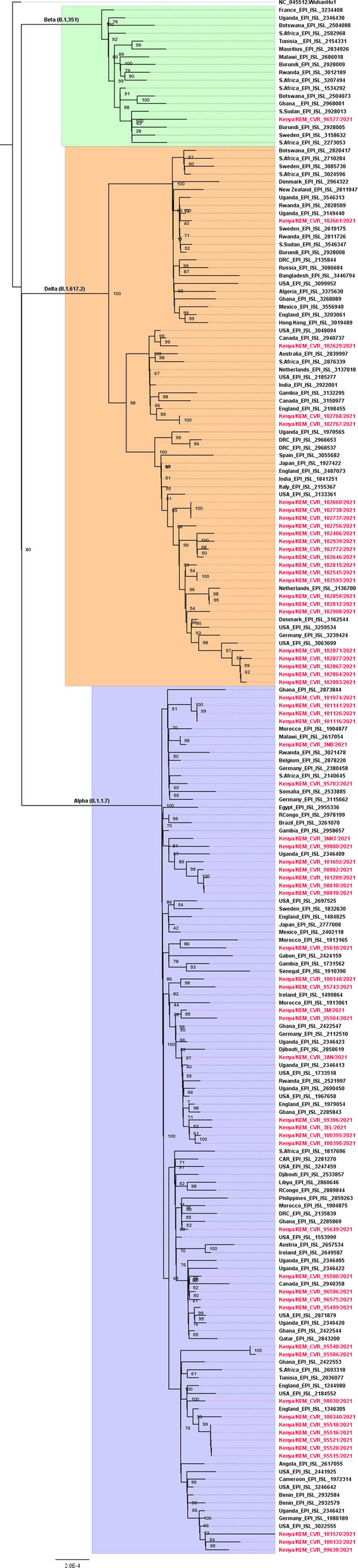
Maximum-likelihood tree of Kenyan and global strains. Phylogenetic analysis depicting assignment of the genome sequences analyzed in the study to SARS-CoV-2 Alpha, Beta, and Delta genetic clades/lineages, signified by different colours. The Kenyan strains are identified in red. The bootstrap numbers at the nodes denote statistical branch support values. The bar indicates the number of nucleotide substitutions per site

Genetic analyses of the SARS-CoV-2 genomes relative to the NC_045512.2-Wuhan-Hu-1 strain revealed a varying degree of amino acid polymorphisms. All the Kenyan sequences contained a D614G substitution in the spike protein-encoding gene. Those belonging to the Alpha (B.1.1.7) lineage displayed 69 -70del and Y144del mutations, while a small proportion (30%) of the Delta (B.1.351) lineage sequences had N354X change. However, these mutations were not peculiar to the Kenyan viruses as they were noted among sequences of global strains available in the GISAID database. Phylogenetic analysis revealed that the local strains of the Alpha and Delta lineages were separated into about 15 and 6 clades of varying sizes, respectively, interspersed among the foreign strains. This interspersion of Kenyan clades among the foreign strains indicates multiple introductions of the viruses from outside Kenya, resulting in local transmission (Fig. 2).

## Discussion

Surveillance of the SARS-CoV-2 by whole genome sequencing is crucial in providing insights into its transmission and evolutionary dynamics [4, 22]. It’s equally valuable in identifying potential variants and virulence determinants [4, 41, 42]. This type of information is critical in evaluating vaccine effectiveness and guiding the design of drugs or diagnostics [41]. In this study, we investigated sixty-four (N = 64) genome sequences of SARS-CoV-2 VOCs in Nairobi and neighboring counties in Kenya between March 2021 and July 2021, utilizing various bioinformatics approaches. Our analyses revealed the circulation of multiple SARS-CoV-2 variants of concern in Nairobi and neighboring counties during the studied period. The Alpha (B.1.1.7) lineage was predominant (65%) from March to May 2021, with Beta (B.1.351) being the minority (< 2%), while Delta (B.1.617) lineage dominated beginning June 2021. This result mirrors findings of related studies conducted elsewhere [43–45] and suggests that the surge in SARS-CoV-2 infection observed in Nairobi and its environs during these periods were largely caused by the introduction and subsequent transmission into the population of the highly transmissible SARS-CoV-2 Alpha (B.1.1.7) and Delta (B.1.617) variants into the population. SARS-CoV-2 community transmissions and the occurrence of different waves of SARS-CoV-2 infection have been documented in Kenya [21, 23]. Moreover, the marked decline in the frequency of virus infection associated with the Alpha (B.1.1.7) variant around May 2021 could possibly be attributed to the public health countermeasures that were instituted to mitigate further seeding of newer virus clusters and the spread of the virus in the population. Overall, our findings indicate that there were multiple introductions of the viruses from outside Kenya, resulting in community transmission that contributed to the large number of new infections observed during the study period. This observation emphasizes the importance of strengthening quarantine and border controls to combat virus imports into Kenya.

As reported elsewhere and as a common feature of the SARS-CoV-2 Alpha, Beta, and Delta lineages [22, 41], all the local strains harbored the D614G mutation in the spike glycoprotein, associated with increased infectivity [9, 46–48]. Interestingly, all of the Alpha (B.1.1.7) viruses in this study had the 69-70del and Y144del deletions in the spike protein, which are linked to increased infectivity and decreased monoclonal antibody binding [49–52]. Furthermore, the H69del and V70del mutations have been associated with a false-negative result for the target S gene in the standard RT-PCR SARS-CoV-2 assay [53, 54]. Similar to the findings of Parvin et al. [4], 30% of the study Delta (B.1.351) viruses had N354X spike mutation, associated with increased virus infectivity [2]. Meanwhile, phylogenetic analysis showed the majority of the Kenyan viruses clustered together in the respective SARS-CoV-2 lineages, except a few that appeared interspersed among the foreign strains. The clustering and interspersion of the Kenyan SARS-CoV-2 genome sequences among those of foreign strains included in the analysis plausibly suggest multiple routes of introduction and transmission into the community.

A limitation of this study was that it did not include samples from all regions of Kenya in order to present a nationwide picture. The other limitation is the relatively small sequence dataset, which may not be representative enough and may have introduced some degree of bias in our findings. However, despite the limitations, we have demonstrated that multiple lineages of SARS-CoV-2 variants of concern (VOC) viz B.1.1.7, B.1.351, and B.1.617 circulated in Nairobi and neighboring counties in Kenya between March and July 2021. These viruses were genetically similar to those that circulated in other regions of the world and are attributable to the different waves of SARS-CoV-2 infection experienced particularly in Nairobi, and Kenya at large. In conclusion, our findings provide a snapshot of information about the SARS-CoV-2 variants co-circulating in Kenya, and more data are needed to construct a more comprehensive picture of the epidemic in Kenya.

## Data Availability

All the SARS-CoV-2 genomes reported in this work have been deposited in the GISAID database under the accession numbers: EPI_ISL_4472133; EPI_ISL_4472540; EPI_ISL_4472541; EPI_ISL_4472846; EPI_ISL_4473129; EPI_ISL_4473132; EPI_ISL_4473149; EPI_ISL_4473150; EPI_ISL_4473153; EPI_ISL_4473226; EPI_ISL_4473242; EPI_ISL_4473270; EPI_ISL_4473302; EPI_ISL_4473307 to EPI_ISL_4473309; EPI_ISL_4473504; EPI_ISL_4473482; EPI_ISL_4473483; EPI_ISL_4474043; EPI_ISL_4474044; EPI_ISL_4474467; EPI_ISL_4474512; EPI_ISL_4474518; EPI_ISL_4474539; EPI_ISL_4474581; EPI_ISL_4474771; EPI_ISL_4505350; EPI_ISL_4505457; EPI_ISL_4505540 to EPI_ISL_4505546; EPI_ISL_4506003; EPI_ISL_4506016; EPI_ISL_4506017; EPI_ISL_4506812; EPI_ISL_4507490; EPI_ISL_4507883; EPI_ISL_4508206; EPI_ISL_4508258; EPI_ISL_4508426; EPI_ISL_4515253; EPI_ISL_4515254; EPI_ISL_4515337 to EPI_ISL_4515340; EPI_ISL_4515415; EPI_ISL_4515456; EPI_ISL_45155537; EPI_ISL_4515844; EPI_ISL_4515845; EPI_ISL_4515707; EPI_ISL_4515900 to EPI_ISL_4515905; EPI_ISL_455616.

## Abbreviations

ACE2: Angiotensin-converting enzyme 2
COVID-19: Coronavirus disease 2019
E gene: Envelope protein gene
GISAID: Global initiative on sharing all influenza data
iTOL: Interactive tree of life
KEMRI: Kenya Medical Research Institute
MoH: Ministry of Health
N gene: Nucleocapsid protein gene
ORF1ab: Open Reading Frame 1ab
PANGOLIN: Phylogenetic Assignment of Named Global Outbreak LiNeages
RBD: Receptor-binding domain
RNA: Ribonucleic acid
RT-PCR: Reverse Transcription Polymerase Chain Reaction
S gene: Spike protein gene
SARS-CoV-2: Severe acute respiratory syndrome coronavirus-2
SERU: Scientific and Ethical Review Unit
SMRF: Sample Management and Repository Facility
VOI: Variants of interest
VOC: Variants of concern
WHO: World Health Organization
